# Exploring the hidden effects: Three-unit metal-ceramic restorations and alveolar bone loss in diabetic patients

**DOI:** 10.6026/9732063002001873

**Published:** 2024-12-31

**Authors:** Abdo Mohammed Mohammed Abdulrazzaq, Ahmed Alassiry, Abdulrahman Ahmed Aseri, Mohammed Hamad Alyami, Sultan Alanazi, Ali Mohammed Ali Alyami, Mahdi Mana Alzamanan, Abdullah Ali Aljari, Mohammed Hussain Mahdi Al-Hutaylah, Abdullah Ali D. Al-mutarid, Abdullah hassan Mohammed AL Khamsan

**Affiliations:** 1 Department of Preventive Dental Science, Najran University, Najran, Saudi Arabia; 2 Department of Prosthodontic Dental Science, Najran University, Najran, Saudi Arabia; 3 Dental intern, Najran University, Najran, Saudi Arabia

**Keywords:** Alveolar bone loss, diabetes mellitus, three-unit fixed posterior metal-ceramic restorations, plaque index, gingival index, periodontal health

## Abstract

The impact of three-unit fixed posterior metal-ceramic restorations on alveolar bone loss in diabetic patients is of interest. Hence, a total of 72 patients at the Najran University, Faculty of Dentistry were divided into two groups: G1 (36 patients, without three-unit fixed posterior metal-ceramic restorations) and G2 (36 patients, with three-unit fixed posterior metal-ceramic restorations). Clinical evaluations and imaging revealed that G2 exhibited significantly higher plaque index (P.I.), gingival index (G.I.) and alveolar bone loss compared to G1 with (p < 0.05). The results underscore the increased risk of periodontal complications and bone loss in diabetic patients with metal-ceramic restorations. This highlights the critical need for enhanced preventive care, including improved oral hygiene practices, careful selection of restorative materials and tailored periodontal monitoring for diabetic patients. Clinicians should consider these factors to reduce the risk of bone resorption and ensure long-term success in restorative dental treatments for this vulnerable population.

## Background:

Diabetes mellitus significantly affects oral health, leading to an increased susceptibility to periodontal diseases and subsequent alveolar bone loss [1]. Diabetic patients are more likely to experience severe periodontal conditions, which are further aggravated by inadequate glycaemic control and the presence of dental restorations [2]. Researchers found that patient with dental restorations exacerbate periodontitis, leading to increased plaque, inflammation and bone loss, particularly in individuals with predisposing factors like diabetes [3]. A prior study found overhanging restorations promote plaque, raising risks of periodontal disease and caries [4]. The relationship between dental restorations and alveolar bone loss is complex and remains an important area of study in periodontal health and dental restorations, including fillings, crowns, implants, dentures, bridges and orthodontic appliances, have been shown to help preserve alveolar bone by replicating the natural tooth structure and providing functional loading that stimulates the surrounding bone [5]. Therefore, it is of interest to compare periodontal health indicators and alveolar bone loss in diabetic patients without and with metal-ceramic restorations.

## Materials and Methods:

The researchers received ethical approval from the University's Research Ethics Committee (Approval No. 202409-076-023599 -052877) and complied with institutional/national guidelines. Clinical assessments followed the ethical standards outlined by the Helsinki Declaration (as revised by the 64th WMA General Assembly, Fortaleza, Brazil, 2013) and subsequent amendments.

## Study groups and data ccollection process:

In this comparative cross-sectional study, 72 diabetic patients were selected from a cohort of 676 at the Faculty of Dentistry, Najran University, Saudi Arabia. The participants, aged 45-60 years, were split into two groups of 36. Group G1 (control) consisted of diabetic patients without three-unit fixed posterior metal-ceramic restorations, while Group G2 (test) included diabetic patients with three-unit fixed posterior metal-ceramic restorations, which had been in place for 8 to 10 years. Clinical evaluations included plaque index (PI) to assess oral hygiene [6], gingival index (GI) to measure gingival inflammation [7] and radiographic analysis to determine alveolar bone loss percentage. The percentage of bone loss was calculated using the formula: ((CEJ-ABC) - 2mm) / ((CEJ-AP) - 2mm) x 100, based on CEJ-ABC and CEJ-AP measurements. For the calculation of the sample size, we utilized the formula developed by Crano and Brewer [8], ensuring methodological rigor and precision that used in medical research,

Where n = Nn*/ N + n*,

The initial estimated sample size (n*) was calculated using the formula n* = P (1 - P)/ (SE) 2, P was assumed to be 0.5 to maximize the sample size, representing the estimated proportion of participants and SE, representing the standard error, was assumed to be 0.05. Patients are stratified by HbA1c levels, with controlled diabetes defined as HbA1c < 7% and uncontrolled diabetes as HbA1c ≥ 7%, reflecting long-term glycaemic control and its health impact [9]. All assessments were performed by two clinician periodontics to ensure consistency in measurements.

## Inclusion:

## The inclusion criteria for selecting the control group G1 of diabetic patients were:

[1] Type 2 diabetes patients' ≥ 2 years.

[2] HbA1c levels ≥ 7%.

[3] Aged 45-60 years.

[4] Without three-unit fixed posterior metal-ceramic restorations.

## The inclusion criteria for selecting the test group G2 of diabetic patients were:

[1] Type 2 diabetes patients' ≥ 2 years.

[2] HbA1c levels ≥ 7%.

[3] Aged 45-60 years.

[4] A minimum of 10 teeth remaining.

[5] With three-unit fixed posterior metal-ceramic restorations and life range 8- 10 years.

## Exclusion criteria:

Patients with significant systemic conditions unrelated to diabetes or those who had undergone periodontal treatment in the last six months were excluded to eliminate potential confounding variables and maintain the focus on the effects of diabetes and metal-ceramic restorations materials on periodontal health.

## Results:

A total of 72 participants were included in the study, with 36 diabetic patients in each group (50%). Control Group 1 (G1) consisted of diabetic patients without three-unit fixed posterior metal-ceramic restorations, while Test Group 2 (G2) included diabetic patients with three-unit fixed posterior metal-ceramic restorations. The goal was to compare the periodontal health of the two groups. Data analysis was performed using SPSS version 18, applying Descriptive Statistics and Two-Sample T-Test. The results showed statistically significant differences between the two groups, with the findings presented in Table 1 and Table 2.

## Statistical analysis and interpretation:

## Age:

Comparable between groups (Control G1: 50.5 ± 4.1 years; Test G2: 51.42 ± 4.87 years).

## Plaque index (P.I.):

High value in Test G2 (2.73 ± 0.14) versus in Control G1 (2.49 ± 0.18) is seen. This is indicating greater plaque accumulation.

## Gingival index (G.I.):

Elevated in Test G2 (2.49 ± 0.18 vs. 2.22 ± 0.04) in Control G1, reflecting increased gingival inflammation.

## HbA1c:

Similar values across groups are suggesting comparable glycaemic control.

## Bone loss (%):

Significantly higher in Test G2 (61.78 ± 6.80%) vs. in Control G1 (44.22 ± 3.12%) is indicating that three-unit fixed posterior metal-ceramic restorations worsen periodontitis, increased plaque, inflammation and bone loss.

## Statistical analysis and interpretation:

## Plaque index (P.I.):

T-statistic: -6.58, p-value: 7.19 x 10-^9^

Significant difference (p < 0.05), with higher values in Test G2 indicating increased plaque accumulation.

## Gingival index (G.I.):

T-statistic: -9.68, p-value: 1.49 x 10-^14^

Significant difference (p < 0.05) with higher values in Test G2 is reflecting greater gingival inflammation.

## HbA1c:

T-statistic: -0.24, p-value: 0.81

No significant difference (p > 0.05), indicating similar glycaemic between groups.

## Bone loss (%):

T-statistic: -14.16, p-value: 3.13 x 10-^22^

Significant difference (p < 0.05), with Test G2 showing substantially greater bone loss indicating that findings highlight potential adverse effects of three-unit fixed posterior metal-ceramic restorations on periodontal health in diabetic patients.

## Discussion:

The analysis demonstrates that diabetic patients with three-unit fixed posterior metal-ceramic restorations exhibit significantly worse oral hygiene, increased gingival inflammation and greater alveolar bone loss compared to those without such restorations because, the roughness and sub-gingival margins of these restorations create niches for plaque accumulation, which harbors pathogenic bacteria. These bacteria trigger immune responses, leading to gingival inflammation and bone loss. Additionally, diabetic patients may experience impaired immune function, exacerbating these inflammatory reactions and this is consistent with previous research highlighting that all diabetic groups showed poorer periodontal conditions, with greater bone loss, higher plaque and gingival index scores, especially in those with class II amalgam restorations or three-unit metal-ceramic prostheses [10]. The findings align with previous studies, indicating that restorative materials may contribute to worsening periodontal problems by promoting plaque buildup and inflammation [11]. The lack of significant difference in HbA1c levels between the two groups indicates that the observed periodontal complications are more closely associated with the restorative treatments rather than overall glucose control [12]. Glycaemic control is vital, but restorative materials directly impact periodontal health, highlighting the need for preventive care, regular monitoring and enhanced oral hygiene in diabetic patients with dental restorations [13]. Another study confirms sub gingival restorations harm periodontal health, with attachment loss becoming clinically evident within 1 to 3 years post-placement [14]. Some researcher found that overhanging restorations lead to plaque retention, gingival inflammation and periodontal tissue destruction and reduced alveolar bone height [15, 16]. Another study investigated the effect of different dental restoration classes, such as crowns and bridge abutment, on patients' periodontal health [17]. A previous study found that metal ceramic prostheses were associated with higher PI, GI scores and probing depths compared to non-abutment teeth and sub gingival crown margins further increased these parameters compared to supragingival margins [18]. Another study found higher alveolar bone loss and more severe periodontitis in diabetic patients, emphasizing the need for increased awareness of the diabetes-oral health link [19]. This innovative analysis underscores the importance of personalized dental care in diabetic patients, focusing on blood sugar control, oral hygiene and material choice to prevent significant alveolar bone loss around restorations. However, the limitations of this study include the sample size of 72 participants restricts the generalizability of the findings; a larger cohort would improve external validity. Furthermore, the cross-sectional design captures only a momentary view of periodontal health, limiting understanding of changes over time. The absence of data on smoking and obesity status, both known to affect periodontal outcomes, is another limitation. Additionally, factors related to the quality of metal-ceramic dental restorations, such as the smoothness of metal-ceramic restorations and sub gingival or supragingival margins and space beneath the pontic, number of units of metal-ceramic dental restorations may have influenced disease progression. Future research should address limitations by increasing sample size and using a longitudinal design to track long-term effects. Incorporating factors like smoking, obesity and medication use would provide a more comprehensive understanding of periodontal health. Additionally, analyzing the quality of metal-ceramic dental restorations (*e.g.*, smoothness, fit and sub-gingival margins) and conducting microbial analysis of plaque could shed light on specific pathogens contributing to inflammation and bone loss, further clarifying the role of restorative materials in periodontal disease.

## Conclusion:

Diabetic individuals with three-unit fixed posterior metal-ceramic restorations exhibit significantly higher Plaque Index, Gingival Index and alveolar bone loss compared to those without such restorations. This highlights the need for rigorous oral hygiene practices and continuous periodontal monitoring in these patients. A proactive yet a preventive approach, including regular assessments, glycaemic control and personalized oral care, to minimize periodontal complications and optimize long-term oral health.

## Figures and Tables

**Table 1:** Descriptive statistics for control G1 and Test G2

**Measure**	**Control G1 (Mean ± Std)**	**Control G1 (Min - Max)**	**Test G2 (Mean ± Std)**	**Test G2(Min - Max)**
Age	50.5 ± 4.1	45 - 60	51.42 ± 4.87	45 - 60
P.I (Plaque Index)	2.49 ± 0.18	2.25 - 2.75	2.73 ± 0.14	2.50 - 3.00
G.I (Gingival Index)	2.22 ± 0.04	2.00 - 2.25	2.49 ± 0.18	2.20 - 2.80
HbA1c	8.78 ± 0.32	8.20 - 9.40	8.73 ± 0.43	8.10 - 9.40
Bone Loss (%)	44.22 ± 3.12	39 - 50	61.78 ± 6.80	52 - 75

**Table 2:** Two-Sample T-Test Results for Control Group 1 (G1) and Test Group 2 (G2)

**Variable**	**t-Statistic**	**P-Value**
Plaque Index (P.I)	-6.58	7.19 x 10^-9^ (p < 0.05).
Gingival Index (G.I)	-9.68	1.49 x 10^-14^ (p < 0.05).
HbA1c	-0.24	0.81 (p > 0.05).
Bone Loss (%)	-14.16	3.13 x 10^-22^ (p < 0.05).

## References

[1] Philip M.P, Bissett S.M (2019). British dental journal..

[2] Christersson L.A (1991). Journal of clinical periodontology..

[3] Abdulrazzaq M, Alanazi S (2024). Bioinformation..

[4] Ercoli C (2021). Journal of Prosthodontics..

[5] Zahn T (2016). Clinical Oral Investigations..

[6] Silness J, Loe H (1964). Acta odontologica Scandinavica..

[7] Loe H, Silness J (1963). Acta Odontol Scand..

[8] Crano W.D (2001). Principles and Methods of Social Research..

[9] Nuha A.E (2023). Diabetes care..

[10] Albakry M (2021). Open Journal of Stomatology..

[11] Abdulrazzaq A.M.M (2024). Cureus..

[12] Dana T.G (2020). Periodontology 2000..

[13] Jasim M.A, Rams E.T (2002). Periodontology 2000..

[14] Schätzle M (2001). Journal of clinical periodontology..

[15] Lang N.P (1983). Journal of clinical periodontology..

[16] Muryani A (2016). Padjadjaran Journal of Dentistry..

[17] Ababnaeh K.T (2011). Oral Health & Preventive Dentistry..

[18] Al-Sinaidi A, Preethanath RS (2014). Saudi Journal of Dental Research..

[19] Tabassum A (2024). Eur J Dent..

